# Stability of Classification Systems for Irritable Bowel Syndrome

**DOI:** 10.1111/apt.70503

**Published:** 2025-12-25

**Authors:** Mais Khasawneh, Vivek C. Goodoory, Cho Ee Ng, Alexander C. Ford, Christopher J. Black

**Affiliations:** ^1^ Leeds Institute of Medical Research at St. James's University of Leeds Leeds UK; ^2^ Leeds Gastroenterology Institute St. James's University Hospital Leeds UK; ^3^ County Durham and Darlington NHS Foundation Trust Durham UK

**Keywords:** irritable bowel syndrome, latent class analysis, psychological burden, stool form, subtype stability

## Abstract

**Background:**

Irritable bowel syndrome (IBS) is a common disorder characterised by recurrent abdominal pain and altered bowel habits. Although IBS is classified according to stool form, symptom patterns fluctuate over time, posing challenges for subtype‐based management.

**Aims:**

To assess stability of IBS classification over 12 months using four approaches: stool form, most troublesome symptom, a seven‐cluster latent class analysis (LCA) incorporating gastrointestinal and psychological symptoms and a simplified LCA based on degree of psychological burden.

**Methods:**

Participants were recruited from ContactME‐IBS, a national UK registry. Individuals meeting Rome IV criteria for IBS completed validated online questionnaires at baseline and 12 months assessing gastrointestinal and psychological symptoms. Participants were sub grouped according to the four classification methods, and stability between baseline and follow‐up was evaluated using Cohen's kappa statistic.

**Results:**

Of 752 participants meeting Rome IV criteria at baseline, 352 completed 12‐month follow‐up, with 259 (73.6%) continuing to meet Rome IV criteria. The highest stability was observed for stool form‐based subtyping (κ = 0.60), particularly in IBS with diarrhoea (83% remained stable). Classification based on psychological burden showed similar stability (κ = 0.54). In contrast, subgrouping by most troublesome symptom (κ = 0.47) and the seven‐cluster LCA (κ = 0.37) demonstrated lower stability.

**Conclusion:**

Different IBS classifications showed only moderate stability over 12 months, highlighting the fluctuating nature of the disorder. Stool form and psychological burden‐based systems were most stable, but no method fully captured IBS variability. Future work could develop dynamic models integrating gastrointestinal and psychological factors to better guide management.

## Graphical abstract

Four classification systems for irritable bowel syndrome were evaluated over 12 months. Stool form and psychological burden‐classification approaches showed the greatest stability, whereas most troublesome symptom and latent class analyses were less stable, emphasising the fluctuating nature of IBS.

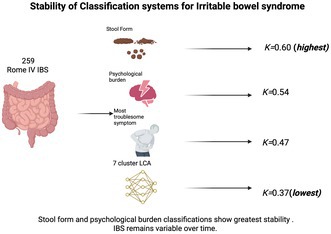

**Abbreviations**
HADS: hospital anxiety and depression scale
IBS: irritable bowel syndrome
IBS‐C: irritable bowel syndrome with constipation
IBS‐D: irritable bowel syndrome with diarrhoea
IBS‐M: irritable bowel syndrome with mixed bowel habits
IBS‐SSS: irritable bowel syndrome severity scoring system
IBS‐U: irritable bowel syndrome unclassified
LCA: latent class analysis
PHQ‐12: patient health questionnaire‐12
VSI: visceral sensitivity index

## 1 Introduction

Irritable bowel syndrome is a common disorder of gut‐brain interaction affecting between 5% and 10% of the global population [1]. It is characterised by recurrent abdominal pain and change in either stool frequency or form [2]. IBS has a considerable impact on healthcare resources, due to management costs [3], society, due to its effect on ability to work [4] and the individual, due to its impact on quality of life and social functioning [4, 5]. The diagnosis is symptom‐based, using the validated Rome IV criteria [6, 7]. These criteria not only aid in diagnosis, but also facilitate subgrouping of patients, based on their stool type according to the Bristol Stool Form Scale, to guide treatment. People with IBS are subgrouped into those with IBS with diarrhoea (IBS‐D), those with IBS with constipation (IBS‐C), those with IBS with mixed bowel habits (IBS‐M) and those with IBS unclassified (IBS‐U), when criteria for the other three subtypes are not met.

Previous studies suggest that these IBS subgroups fluctuate over time [8, 9, 10]. More than 50% of patients have been reported to change subgroup over 1 year [8, 11, 12]. This instability presents challenges, as therapeutic approaches are tailored to the predominant abnormality in stool form. In addition, in clinical practice many physicians ask patients with IBS what the most troublesome of their symptoms is, such as abdominal pain, bloating, constipation, diarrhoea or urgency and direct drug therapy towards this. Furthermore, the complex heterogeneous nature of IBS goes beyond stool form alone, as mood and psychological health play an important role in the development and persistence of symptoms [13, 14, 15, 16, 17]. We and others, have shown using latent class analysis (LCA) that there are clusters of individuals with IBS in whom gastrointestinal symptoms predominate, clusters in whom gastrointestinal symptoms are milder and psychological symptoms predominate, and clusters in whom gastrointestinal symptoms are more severe and are part of a broader picture, including symptoms of anxiety, depression or somatisation [18, 19, 20, 21, 22].

We hypothesised that other approaches to subgrouping people with IBS, using most troublesome symptom or novel cluster‐based approaches to subgrouping IBS based on a combination of gastrointestinal and psychological symptoms, would demonstrate more stability over time. If demonstrated in this study, these could serve as useful alternatives for guiding therapeutic decision making.

## 2 Methods

We recruited participants from ContactME‐IBS, a national UK registry of individuals with IBS who have expressed interest in participating in research studies [23]. The registry is managed by County Durham and Darlington NHS Foundation Trust and recruits participants across the UK via advertisements in primary care settings, hospital clinics, pharmacies and social media platforms. Individuals join the registry by completing a brief questionnaire regarding their bowel symptoms and providing contact information. We have published previous data from this cohort [3, 5]. An electronic invitation to participate in the study was sent to all registered individuals in July 2021. The only exclusion criterion was an inability to understand written English. Responses were collected through an online survey platform, and a reminder email was sent to non‐responders in August 2021. Respondents to the baseline questionnaire were invited by electronic mailshot to participate in the follow‐up survey 12 months later in July 2022. Those who did not respond to the follow‐up survey received a reminder email in August 2022. To encourage participation, respondents were entered into a prize draw for one of three gift cards (£200, £100 or £50) at baseline and follow‐up. Ethical approval for the baseline survey was granted in March 2021 (reference MREC 20–051) and for the follow‐up survey in April 2022 (reference MREC 20–051 Amendment 1) by the University of Leeds Research Ethics Committee.

### 2.1 Data Collection and Synthesis

#### 2.1.1 Demographic and Symptom Data

All questionnaire data, both at baseline and 12 months, were collected electronically. We recorded demographic data, including age, sex, ethnicity, marital status, educational attainment, annual income and lifestyle factors including tobacco and alcohol consumption. The presence of IBS was defined according to the Rome IV criteria using the validated questionnaire [24], with classification based on the recommended scoring algorithm [2]. Participants were asked to self‐report the duration of their IBS symptoms and whether their IBS developed after an acute enteric infection, as well as whether or not they had seen a primary care physician or a gastroenterologist with IBS symptoms in the prior 12 months.

#### 2.1.2 IBS Symptom Severity, Mood and Somatic Symptoms and Gastrointestinal Symptom‐specific Anxiety

We evaluated symptom severity using the IBS severity scoring system (IBS‐SSS) [25], which has a maximum score of 500 points; < 75 points indicates remission, 75–174 points mild, 175–299 points moderate and 300–500 points severe symptoms. We assessed anxiety and depression using the hospital anxiety and depression scale (HADS) [26]. The total score ranges from 0 to 21 for each domain. We classified severity as normal (score 0–7), borderline (8–10) or abnormal (≥ 11). We evaluated the somatic symptom burden using the Patient Health Questionnaire‐12 (PHQ‐12) [27], which is derived from the validated PHQ‐15 [28]. PHQ‐12 scores range from 0 to 24, with symptom severity classified as high (≥ 13), moderate (8–12), low (4–7) or minimal (0–3). Gastrointestinal symptom‐specific anxiety was assessed using the Visceral Sensitivity Index (VSI) [29], a 15‐item instrument with responses rated on a 6‐point scale ranging from ‘strongly disagree’ (score 0) to ‘strongly agree’ (score 5). As no validated cut‐off values exist to categorise levels of gastrointestinal‐specific anxiety, VSI scores were divided into three equally sized groups to represent low, medium and high levels of gastrointestinal‐specific anxiety.

### 2.2 Subgrouping of Participants

Participants were subgrouped in four different ways at both baseline and 12‐month follow‐up. Firstly, IBS subtypes were determined based on their reporting of abnormal stools, as defined by the Bristol stool form scale [30], on the days on which stools were abnormal, as recommended by the Rome IV questionnaire [24], Secondly, participants identified their most troublesome symptom from five options: abdominal pain, constipation, diarrhoea, abdominal bloating or urgency. Thirdly, we used our previously validated LCA to assign participants into the seven unique clusters, as we have described previously [18], according to the reporting of both gastrointestinal and psychological symptoms, using syntax previously saved for this purpose. The variables used from the Rome IV diagnostic questionnaire, HADS and PHQ‐12 to assign cluster membership are provided in (Table S1). The seven clusters, as detailed in (Figure S1), include diarrhoea and urgency with low psychological burden (cluster 1, Figure S1 A), diarrhoea, abdominal pain and urgency with high psychological burden (cluster 4, Figure S1 D), constipation and bloating with low psychological burden (cluster 7, Figure S1 G), constipation, abdominal pain and bloating with high psychological burden (cluster 5, Figure S1 E), low overall gastrointestinal symptom severity with low psychological burden (cluster 3, Figure S1 C), low bowel symptom severity with abdominal pain and high psychological burden (cluster 2, Figure S1 B), and high overall gastrointestinal symptom severity with high psychological burden (cluster 6, Figure S1 F). Finally, participants were assigned into clusters based on the degree of psychological burden, again as we have done in previous studies [31]. As described, there are four clusters with a high psychological burden (clusters 2, 4–6) and three with a low psychological burden (clusters 1, 3 and 7).

### 2.3 Statistical Analysis

In our primary analyses we included only participants who met the Rome IV criteria for IBS at both baseline and 12‐month follow‐up. We compared characteristics of individuals with Rome IV IBS at baseline who were followed up successfully at 12 months with those of individuals with Rome IV IBS at baseline who were not followed up using a *χ^2^* for categorical variables and an independent samples *t*‐test for continuous data. We then compared the proportions of those participants who were successfully followed up at 12 months and who met Rome IV criteria for IBS at both baseline and 12‐month follow‐up who transitioned to a different IBS subgroup at 12 months according to the four different approaches to classifying IBS. The degree of agreement for each of the four approaches to subgrouping between baseline and follow‐up was assessed using Cohen's Kappa statistic, where values of 0–0.20 indicate slight agreement, 0.21–0.40 fair agreement, 0.41–0.60 moderate agreement, 0.61–0.80 substantial agreement and 0.81–1 almost perfect agreement. We performed sensitivity analyses including all individuals with Rome IV IBS who were followed up successfully at 12 months. All statistical analyses were performed using SPSS for Windows (version 30.0; SPSS Inc., Chicago, IL).

## 3 Results

Of 4280 registered individuals, 1278 (29.9%) completed the baseline questionnaire. Among these, 752 participants (58.8%) met Rome IV criteria for IBS at baseline (mean age 45.3 years, 655 (87.1%) female). At 12 months, 352 (46.8%) of these 752 individuals with Rome IV IBS were followed up successfully. Demographic details of the 352 individuals with Rome IV IBS at baseline followed up successfully and the 400 individuals with Rome IV IBS at baseline who were not followed up are provided in Table S2. Those successfully followed up were older and less likely to be smokers, but there were no other significant differences, including according to the four different approaches to classifying IBS. Of the 352 individuals with Rome IV IBS at baseline, 259 (73.6%) still met the Rome IV criteria for IBS at 12 months. These 259 individuals formed the population of interest for our primary analyses, with data from all 352 individuals meeting the Rome IV criteria at study inclusion included in the sensitivity analyses.

### 3.1 Stability of IBS According to Bristol Stool Form Scale

The highest level of stability was observed using this classification (Kappa statistic = 0.60), with 193 (74.5%) participants reporting the same abnormality of stools, according to the Bristol stool form scale, at 12‐month follow‐up (Table 1). IBS‐C was the least stable, with 67.4% of 42 individuals with IBS‐C at baseline remaining in this subtype, 27.9% transitioning to IBS‐M and only 2.3% to either IBS‐D or IBS‐U at follow‐up. Of the 111 classified as having IBS‐D at baseline, 83.0% remained in this subtype, while 12.3% were reclassified as IBS‐M and only 3.8% and 0.9% as IBS‐C or IBS‐U, respectively. Among 104 individuals with IBS‐M at baseline, 71.7% remained classified as IBS‐M, 19.8% transitioned to IBS‐D and only 8.5% to IBS‐C. Among four individuals with IBS‐U at baseline, 75.0% had IBS‐M at follow‐up and 25.0% IBS‐D. When we included data from all 352 individuals with Rome IV IBS at baseline, results were similar with 255 (72.4%) participants reporting the same abnormality of stools and a Kappa statistic of 0.57 (Table S3).

**TABLE 1:** Stability of IBS according to the Bristol stool form scale in 259 individuals with Rome IV IBS at baseline and 12‐month follow‐up.

	IBS‐C at 12‐month follow‐up (%)	IBS‐D at 12‐month follow‐up (%)	IBS‐M at 12‐month follow‐up (%)	IBS‐U at 12‐month follow‐up (%)
IBS‐C at baseline (*n* = 42)	29 (67.4)	1 (2.3)	12 (27.9)	1 (2.3)
IBS‐D at baseline (*n* = 111)	4 (3.8)	88 (83.0)	13 (12.3)	1 (0.9)
IBS‐M at baseline (*n* = 104)	9 (8.5)	21 (19.8)	76 (71.7)	0 (0.0)
IBS‐U at baseline (*n* = 2)	0 (0.0)	1 (25.0)	3 (75.0)	0 (0.0)

*Note:* Kappa = 0.60.

### 3.2 Stability of IBS According to Most Troublesome Symptom

In terms of most troublesome symptom, 153 (59.1%) participants continued to report the same most troublesome symptom at 12 months (Kappa statistic = 0.47). Among 57 individuals who initially identified abdominal pain as their most troublesome symptom, 63.2% continued to report abdominal pain as their most troublesome symptom at follow‐up, with the largest proportion (15.8%) of those who fluctuated reporting bloating or distension at 12 months (Table 2). Subjects with constipation as their most troublesome symptom showed the greatest degree of stability, with 82.4% of the 17 reporting this as their most troublesome symptom at baseline continuing to report constipation at 12 months, and again the largest proportion who fluctuated reporting bloating or distension (11.8%). Among the 41 participants who reported diarrhoea as their most troublesome symptom at baseline, 51.2% continued to report diarrhoea at follow‐up, with the largest proportion who fluctuated reporting urgency (36.6%). Of the 71 participants who reported bloating or distension as their most troublesome symptom at baseline, 63.4% continued to report bloating or distension at 12 months, with the largest proportion of those who fluctuated reporting abdominal pain (18.3%). Finally, of those who initially reported urgency as their most troublesome symptom, 50.7% continued to report urgency at follow‐up, with the largest proportion of those who fluctuated reporting diarrhoea (23.3%). In sensitivity analysis including data from all 352 individuals with Rome IV IBS at baseline, 210 (59.7%) participants reported the same most troublesome symptom (Kappa statistic = 0.48) (Table S4).

**TABLE 2:** Stability of IBS according to most troublesome symptom in 259 individuals with Rome IV IBS at baseline and 12‐month follow‐up.

	Abdominal pain at 12‐month follow‐up (%)	Constipation at 12‐month follow‐up (%)	Diarrhoea at 12‐month follow‐up (%)	Bloating or distension at 12‐month follow‐up (%)	Urgency at 12‐month follow‐up (%)
Abdominal pain at baseline (*n* = 57)	36 (63.2)	3 (5.3)	6 (10.5)	9 (15.8)	3 (5.3)
Constipation at baseline (*n* = 17)	1 (5.9)	14 (82.4)	0 (0.0)	2 (11.8)	0 (0.0)
Diarrhoea at baseline (*n* = 41)	2 (4.9)	0 (0.0)	21 (51.2)	3 (7.3)	15 (36.6)
Bloating or distension at baseline (*n* = 71)	13 (18.3)	4 (5.6)	3 (4.2)	45 (63.4)	6 (8.5)
Urgency at baseline (*n* = 73)	7 (9.6)	2 (2.7)	17 (23.3)	10 (13.7)	37 (50.7)

*Note:* Kappa = 0.47.

### 3.3 Stability of IBS According to Clusters Based on Both Gastrointestinal and Psychological Symptoms

When we applied our LCA model, based on both gastrointestinal and psychological symptoms, to the participants, 124 (47.9%) remained in the same cluster at follow‐up (Kappa statistic = 0.37). The most stable clusters were clusters 3 and 6, with 54.1% and 56.0%, respectively, remaining in those clusters (Table 3). Among 56 individuals in cluster 1 at baseline, with diarrhoea and urgency and low psychological burden, 42.9% remained in cluster 1. These individuals were most likely to fluctuate to either cluster 3 (23.2%), with low overall gastrointestinal symptom severity with low psychological burden, or cluster 4 (17.9%), with diarrhoea, abdominal pain and urgency with high psychological burden. In 64 individuals in cluster 2 at baseline, with low bowel symptom severity with abdominal pain and high psychological burden, 45.3% remained in cluster 2. These individuals were most likely to fluctuate into cluster 4 (14.1%), with diarrhoea, abdominal pain and urgency with high psychological burden. Among 37 in cluster 3 at baseline, with low overall gastrointestinal symptom severity with low psychological burden, 54.1% remained in cluster 3, with the highest proportion fluctuating to cluster 1 (29.7%), with diarrhoea and urgency with low psychological burden. In 51 individuals in cluster 4 at baseline, with diarrhoea, abdominal pain and urgency with high psychological burden, 49.0% remained in cluster 4. These individuals were most likely to fluctuate to cluster 6 (13.7%), with high overall gastrointestinal symptom severity with high psychological burden. Among 10 individuals in cluster 5 at baseline, with constipation, abdominal pain and bloating with high psychological burden, 50.0% remained in cluster 5. The largest proportion of these individuals fluctuated to cluster 2 (30.0%), with low bowel symptom severity with abdominal pain and high psychological burden. In 25 individuals in cluster 6 at baseline, with high overall gastrointestinal symptom severity with high psychological burden, 56.0% remained in cluster 6. These individuals were most likely to fluctuate to cluster 2 (24.0%), with low bowel symptom severity with abdominal pain and high psychological burden. Finally, among 16 individuals in cluster 7 at baseline, with constipation and bloating with low psychological burden, 43.8% remained in cluster 7. The highest proportion of these individuals fluctuated to either cluster 1 (18.8%), with diarrhoea and urgency with low psychological burden or cluster 3 (18.8%), with low overall gastrointestinal symptom severity with low psychological burden. Therefore, among the seven clusters, clusters 3 and 6 were the most stable and clusters 1 and 7 the least stable. When we included data from all 352 individuals with Rome IV IBS at baseline, results were similar with 176 (50.0%) participants reporting the same abnormality of stools and a Kappa statistic of 0.39 (Table S5).

**TABLE 3:** Stability of IBS according to clusters based on both gastrointestinal and psychological symptoms in 259 individuals with Rome IV IBS at baseline and 12‐month follow‐up.

	Cluster 1: Diarrhoea and urgency with low psychological burden at 12‐month follow‐up (%)	Cluster 2: Low bowel symptom severity with abdominal pain and high psychological burden at 12‐month follow‐up (%)	Cluster 3: Low overall gastrointestinal symptom severity with low psychological burden at 12‐month follow‐up (%)	Cluster 4: Diarrhoea, abdominal pain and urgency with high psychological burden at 12‐month follow‐up (%)	Cluster 5: Constipation, abdominal pain and bloating with high psychological burden at 12‐month follow‐up (%)	Cluster 6: High overall gastrointestinal symptom severity with high psychological burden at 12‐month follow‐up (%)	Cluster 7: Constipation and bloating with low psychological burden at 12‐month follow‐up (%)
Cluster 1: Diarrhoea and urgency with low psychological burden at baseline (*n* = 56)	24 (42.9)	7 (12.5)	13 (23.2)	10 (17.9)	0 (0.0)	1 (1.8)	1 (1.8)
Cluster 2: Low bowel symptom severity with abdominal pain and high psychological burden at baseline (*n* = 64)	8 (12.5)	29 (45.3)	8 (12.5)	9 (14.1)	2 (3.1)	4 (6.3)	4 (6.3)
Cluster 3: Low overall gastrointestinal symptom severity with low psychological burden at baseline (*n* = 37)	11 (29.7)	4 (10.8)	20 (54.1)	1 (2.7)	0 (0.0)	0 (0.0)	1 (2.7)
Cluster 4: Diarrhoea, abdominal pain and urgency with high psychological burden at baseline (*n* = 51)	6 (11.8)	5 (9.8)	5 (9.8)	25 (49.0)	2 (3.9)	7 (13.7)	1 (2.0)
Cluster 5: Constipation, abdominal pain and bloating with high psychological burden at baseline (*n* = 10)	0 (0.0)	3 (30.0)	0 (0.0)	1 (10.0)	5 (50.0)	1 (10.0)	0 (0.0)
Cluster 6: High overall gastrointestinal symptom severity with high psychological burden at baseline (*n* = 25)	0 (0.0)	6 (24.0)	0 (0.0)	3 (12.0)	2 (8.0)	14 (56.0)	0 (0.0)
Cluster 7: Constipation and bloating with low psychological burden at baseline (*n* = 16)	3 (18.8)	2 (12.5)	3 (18.8)	1 (6.3)	0 (0.0)	0 (0.0)	7 (43.8)

*Note:* Kappa = 0.37.

### 3.4 Stability of IBS According to Clusters Based on the Degree of Psychological Burden

When we examined stability according to whether individuals were in a high or low psychological burden cluster at baseline, 201 (77.6%) remained in a cluster with the same degree of psychological burden at 12‐month follow‐up (Kappa statistic = 0.54). Among the 109 participants in a cluster with a low psychological burden at baseline, 76.1% remained in one of these clusters at follow‐up (Table 4). Of the 150 in a cluster with a high psychological burden at baseline, 78.7% remained in a cluster with a high psychological burden. In sensitivity analysis including data from all 352 individuals with Rome IV IBS at baseline, 267 (75.8%) participants reported the same most troublesome symptom (Kappa statistic = 0.51) (Table S6).

**TABLE 4:** Stability of IBS according to clusters based on the degree of psychological burden in 259 individuals with Rome IV IBS at baseline and 12‐month follow‐up.

	Low psychological burden cluster at 12‐month follow‐up (%)	High psychological burden cluster at 12‐month follow‐up (%)
Low psychological burden cluster at baseline (*n* = 109)	83 (76.1)	26 (23.9)
High psychological burden cluster at baseline (*n* = 150)	32 (21.3)	118 (78.7)

*Note:* Kappa = 0.54.

## 4 Discussion

We assessed the stability of IBS in this longitudinal follow‐up study across four different classification methods: stool form, most troublesome symptom, our LCA consisting of seven different clusters based on degree of gastrointestinal and psychological symptoms and a simplification of our LCA based on whether clusters were of high or low psychological burden. The classification system based on stool form demonstrated the highest degree of stability over the 12‐month follow‐up period, particularly among participants with IBS‐D, with over 80% remaining in the same subgroup, followed by IBS‐M and IBS‐C. Classification based on whether clusters were of high or low psychological burden demonstrated relatively similar stability and this was the classification system with the most individuals remaining in the same subgroup during follow‐up. In contrast, subgrouping systems based on either most troublesome symptom or the seven‐cluster LCA model demonstrated the least stability, with the seven clusters demonstrating only fair agreement between baseline and 12‐month follow‐up. Results were very similar when we included all individuals with Rome IV IBS at baseline, irrespective of whether they met the Rome IV criteria at 12‐month follow‐up. Although our results suggest that the subgrouping system based on stool form may offer a relatively more stable framework for predicting the natural history of IBS over time, it is important to point out that none of the approaches to subgrouping IBS led to more than moderate agreement between baseline and 12 months, highlighting the dynamic nature of IBS symptomatology, with considerable fluctuation over a 12‐month period.

Our study recruited a large number of participants who met Rome IV criteria. The sample was diverse, including individuals across a wide range of age groups, educational backgrounds, income levels and relationship statuses. Participants also reflected varied healthcare experiences, from those who had never sought medical attention to those managed in primary care or who had consulted a gastroenterologist. This diversity enhances the representativeness of our findings for people living with IBS in the UK. Participants were recruited based on a self‐reported diagnosis of IBS from a UK national registry, thereby broadening recruitment beyond conventional sources within primary, secondary or tertiary care. Furthermore, we obtained near‐complete data for the variables of interest by using validated online questionnaires with mandatory fields at follow‐up.

The absence of access to participants’ medical records is a limitation and precluded the definitive exclusion of alternative or coexisting, conditions, such as coeliac disease, bile acid diarrhoea or inflammatory bowel disease, which may present with overlapping symptoms [32, 33, 34]. However, IBS is considerably more prevalent than these conditions. Although national UK clinical guidelines recommend that such disorders be evaluated or excluded during the diagnostic assessment of IBS [35, 36], nearly 90% of individuals registered with ContactME‐IBS reported having consulted a primary care physician or gastroenterologist regarding their IBS symptoms. Collectively, these factors provide reasonable assurance that the recruited cohort predominantly comprised individuals with confirmed or highly probable IBS. However, we also do not know what clinical course or treatment was followed (if any) in the 12‐month follow‐up period and this is another limitation.

Previous research has examined the stability of IBS diagnoses and subtypes [8, 9, 37, 38, 39, 40, 41]. However, most available data are derived from population‐based cross‐sectional studies or referral cohorts, limiting the generalisability of their findings. Consequently, the longitudinal stability of IBS subtypes in large, well‐characterised cohorts remains poorly understood. Although our group and others have compared subtype stability using different diagnostic criteria [10, 12, 42], few studies have incorporated psychological factors to evaluate their influence on the long‐term course of the disorder. This study is the first, to our knowledge, to examine IBS subtype stability using multiple classification approaches that extend beyond traditional stool consistency and form.

Our results confirm that IBS symptomatology is relatively unstable across all classification methods used. Subtyping based on stool form, particularly among individuals with IBS‐D, demonstrated the greatest stability although, interestingly, the degree of stability of the ‘construct’ of IBS‐C, based on the Bristol stool form scale, was less stable than the ‘concept’ of constipation itself, when reported as the most troublesome symptom. The relative consistency of psychological burden‐based classifications suggests that this approach may also provide a reasonably stable representation of IBS symptom patterns over time. However, the limited agreement across all systems highlights the fluctuating nature of IBS and the challenges of using static subtyping to predict its clinical course.

Indeed, perhaps it is the wrong approach to prioritise stability as the principal measure for defining a good classification system for IBS, given its heterogenous nature. The biopsychosocial model is key to the management of IBS and serves to highlight the contrasting dimensions of a patient's illness experience and the need for holistic care. Although gastrointestinal symptoms and psychological health may change in tandem, with improvements in one correlating with improvements in the other or vice versa, in keeping with our understanding of the gut‐brain axis in IBS, they may also change independently. This is highlighted by the results of the LCA model, where psychological burden, whether high or low, was more likely to remain stable despite fluctuations in the pattern of gastrointestinal symptoms. Equally, there could be improvement in gastrointestinal symptom severity overall, even though the specific nature of those symptoms has changed over time; for example, a bowel habit that has fluctuated from diarrhoea to constipation but is less bothersome to the patient in general. IBS classification systems that focus only on stool form or predominant symptom fail to capture these nuances, irrespective of whether they remain stable over time. Overall, our findings emphasise the need for a more dynamic approach that integrates gastrointestinal and psychological factors at more than one point in time to support adaptive management strategies for patients with IBS.

In conclusion, this longitudinal follow‐up study evaluated the 12‐month stability of IBS classification according to four methods: stool form, most troublesome symptom, a seven‐cluster LCA, incorporating gastrointestinal and psychological symptoms and a simplified LCA based on degree of psychological burden in the seven clusters. Classifications based on stool form and degree of psychological burden demonstrated greatest stability and may provide a more stable assessment of symptoms over time, compared with other methods. However, all approaches showed only fair to moderate agreement over time, emphasising the fluctuating nature of IBS. Future research should focus on developing dynamic classification systems that integrate both gastrointestinal and psychological factors to better capture the variable course of IBS.

## Author Contributions

M.K., V.C.G., C.E.N., A.C.F., and C.J.B. conceived and drafted the study. V.C.G. and C.E.N. collected all data. M.K., C.J.B., and A.C.F. analyzed and interpreted the data. M.K. drafted the manuscript. All authors have approved the final draft of the manuscript.

## Funding

Unrestricted research monies were provided by Tillotts Pharma UK Ltd. The funder had no input into the concept, design, analysis or reporting of the study.

## Conflicts of Interest

Mais Khasawneh: none. Vivek C. Goodoory: none. Cho Ee Ng: none. Alexander C. Ford: none. Christopher J. Black: none.

## Supporting information


**Table S1:** Variables used to assign cluster membership in the latent class analysis.
**Table S2:** Demographic data, psychological characteristics and classification of individuals with Rome IV‐defined IBS at baseline followed up successfully at 12 months versus individuals with Rome IV‐defined IBS at baseline not followed up at 12 months.
**Table S3:** Stability of IBS according to the Bristol stool form scale in 352 individuals with Rome IV IBS at baseline.
**Table S4:** Stability of IBS according to most troublesome symptom in 352 individuals with Rome IV IBS at baseline.
**Table S5:** Stability of IBS according to clusters based on both gastrointestinal and psychological symptoms in 352 individuals with Rome IV IBS at baseline.
**Table S6:** Stability of IBS according to clusters based on the degree of psychological burden in 352 individuals with Rome IV IBS at baseline.
**Figure S1:** Profiles of the seven clusters.

## Data Availability

The data that support the findings of this study are available from the corresponding author upon reasonable request.

## References

[1] P. Oka , H. Parr , B. Barberio , C. J. Black , E. V. Savarino , and A. C. Ford , “Global Prevalence of Irritable Bowel Syndrome According to Rome III or IV Criteria: A Systematic Review and Meta‐Analysis,” Lancet Gastroenterology & Hepatology 5, no. 10 (2020): 908–917. 32702295 10.1016/S2468-1253(20)30217-X

[2] F. Mearin , B. E. Lacy , L. Chang , et al., “Bowel Disorders,” Gastroenterology 150, no. 6 (2016): 1393–1407.e5. 10.1053/j.gastro.2016.02.031 27144627

[3] V. C. Goodoory , C. E. Ng , C. J. Black , and A. C. Ford , “Direct Healthcare Costs of Rome IV or Rome III‐Defined Irritable Bowel Syndrome in The United Kingdom,” Alimentary Pharmacology and Therapeutics 56, no. 1 (2022): 110–120. 35491477 10.1111/apt.16939 PMC9325446

[4] A. Frandemark , H. Tornblom , S. Jakobsson , and M. Simren , “Work Productivity and Activity Impairment in Irritable Bowel Syndrome (IBS): A Multifaceted Problem,” American Journal of Gastroenterology 113 (2018): 1540–1549. 30254230 10.1038/s41395-018-0262-x

[5] V. C. Goodoory , E. A. Guthrie , C. E. Ng , C. J. Black , and A. C. Ford , “Factors Associated With Lower Disease‐Specific and Generic Health‐Related Quality of Life in Rome IV Irritable Bowel Syndrome,” Alimentary Pharmacology and Therapeutics 57, no. 3 (2023): 323–334. 36544055 10.1111/apt.17356

[6] P. M. Hellstrom and P. Benno , “The Rome IV: Irritable Bowel Syndrome ‐ A Functional Disorder,” Best Practice & Research. Clinical Gastroenterology 40‐41 (2019): 101634. 10.1016/j.bpg.2019.101634 31594650

[7] C. J. Black , O. Craig , D. J. Gracie , and A. C. Ford , “Comparison of the Rome IV Criteria With The Rome III Criteria For The Diagnosis of Irritable Bowel Syndrome in Secondary care,” Gut 70, no. 6 (2021): 1110–1116. 32973070 10.1136/gutjnl-2020-322519

[8] D. A. Drossman , C. B. Morris , Y. Hu , et al., “A Prospective Assessment of Bowel Habit in Irritable Bowel Syndrome in Women: Defining an Alternator,” Gastroenterology 128, no. 3 (2005): 580–589. 15765393 10.1053/j.gastro.2004.12.006

[9] O. S. Palsson , J. S. Baggish , M. J. Turner , and W. E. Whitehead , “IBS Patients Show Frequent Fluctuations Between Loose/Watery and Hard/Lumpy Stools: Implications for Treatment,” American Journal of Gastroenterology 107, no. 2 (2012): 286–295. 22068664 10.1038/ajg.2011.358 PMC3855407

[10] B. Barberio , L. A. Houghton , Y. Yiannakou , E. V. Savarino , C. J. Black , and A. C. Ford , “Symptom Stability in Rome IV vs Rome III Irritable Bowel Syndrome,” American Journal of Gastroenterology 116, no. 2 (2021): 362–371. 33009062 10.14309/ajg.0000000000000946

[11] V. Garrigues , F. Mearin , X. Badía , et al., “Change Over Time of Bowel Habit in Irritable Bowel Syndrome: A Prospective, Observational, 1‐year Follow‐up Study (RITMO study),” Alimentary Pharmacology and Therapeutics 25, no. 3 (2007): 323–332. 17217445 10.1111/j.1365-2036.2006.03197.x

[12] S. D. Dorn , C. B. Morris , Y. Hu , et al., “Irritable Bowel Syndrome Subtypes Defined by Rome II and Rome III Criteria are Similar,” Journal of Clinical Gastroenterology 43, no. 3 (2009): 214–220. 19623100 10.1097/MCG.0b013e31815bd749

[13] N. A. Koloski , P. M. Boyce , and N. J. Talley , “Somatization an Independent Psychosocial Risk Factor for Irritable Bowel Syndrome But Not Dyspepsia: A Population‐Based Study,” European Journal of Gastroenterology & Hepatology 18, no. 10 (2006): 1101–1109. 16957517 10.1097/01.meg.0000231755.42963.c6

[14] N. A. Koloski , M. Jones , J. Kalantar , M. Weltman , J. Zaguirre , and N. J. Talley , “The Brain–Gut Pathway in Functional Gastrointestinal Disorders is Bidirectional: A 12‐year Prospective Population‐Based Study,” Gut 61, no. 9 (2012): 1284–1290. 22234979 10.1136/gutjnl-2011-300474

[15] N. Koloski , M. Jones , and N. Talley , “Evidence That Independent Gut‐to‐Brain and Brain‐to‐Gut Pathways Operate in The Irritable Bowel Syndrome and Functional Dyspepsia: A 1‐year Population‐Based Prospective Study,” Alimentary Pharmacology and Therapeutics 44, no. 6 (2016): 592–600. 27444264 10.1111/apt.13738

[16] M. P. Jones , J. Tack , L. Van Oudenhove , et al., “Mood And Anxiety Disorders Precede Development of Functional Gastrointestinal Disorders in Patients But Not in the Population,” Clinical Gastroenterology and Hepatology 15, no. 7 (2017): 1014–1020.e4. 28087404 10.1016/j.cgh.2016.12.032

[17] E. Clevers , J. Tack , H. Törnblom , et al., “Development of Irritable Bowel Syndrome Features Over a 5‐Year Period,” Clinical Gastroenterology and Hepatology 16, no. 8 (2018): 1244–1251.e1241. 29510214 10.1016/j.cgh.2018.02.043

[18] C. J. Black , Y. Yiannakou , E. A. Guthrie , R. West , L. A. Houghton , and A. C. Ford , “A Novel Method to Classify and Subgroup Patients With IBS Based on Gastrointestinal Symptoms and Psychological Profiles,” American Journal of Gastroenterology 116, no. 2 (2021): 372–381. 33110014 10.14309/ajg.0000000000000975

[19] C. J. Black , L. A. Houghton , R. M. West , et al., “Novel Irritable Bowel Syndrome Subgroups Are Reproducible in the Global Adult Population,” Clinical Gastroenterology and Hepatology 23, no. 6 (2025): 1039–1048.e1037. 38876193 10.1016/j.cgh.2024.05.042

[20] A. V. Polster , O. S. Palsson , H. Törnblom , et al., “Subgroups of IBS Patients are Characterized by Specific, Reproducible Profiles of GI and non‐GI Symptoms and Report Differences in Healthcare Utilization: A Population‐Based Study,” Neurogastroenterology and Motility 31, no. 1 (2019): e13483. 30393924 10.1111/nmo.13483

[21] A. Byale , R. J. Lennon , S. Byale , et al., “High‐Dimensional Clustering of 4000 Irritable Bowel Syndrome Patients Reveals Seven Distinct Disease Subsets,” Clinical Gastroenterology and Hepatology 22, no. 1 (2024): 173–184.e112. 36174942 10.1016/j.cgh.2022.09.019 PMC10040474

[22] A. Polster , L. Van Oudenhove , M. Jones , L. Ohman , H. Tornblom , and M. Simren , “Mixture Model Analysis Identifies Irritable Bowel Syndrome Subgroups Characterised by Specific Profiles of Gastrointestinal, Extraintestinal Somatic and Psychological Symptoms,” Alimentary Pharmacology and Therapeutics 46, no. 5 (2017): 529–539. 28671338 10.1111/apt.14207

[23] County Durham and Darlington NHS Foundation Trust , “ContactME‐IBS [Internet],” (2021), https://www.contactme‐ibs.co.uk.

[24] O. S. Palsson , W. E. Whitehead , M. A. van Tilburg , et al., “Rome IV Diagnostic Questionnaires and Tables for Investigators and Clinicians,” Gastroenterology 150, no. 6 (2016): 1481–1491. 10.1053/j.gastro.2016.02.014 27144634

[25] C. Y. Francis , J. Morris , and P. J. Whorwell , “The Irritable Bowel Severity Scoring System: A Simple Method of Monitoring Irritable Bowel Syndrome and its Progress,” Alimentary Pharmacology and Therapeutics 11, no. 2 (1997): 395–402. 9146781 10.1046/j.1365-2036.1997.142318000.x

[26] A. S. Zigmond and R. P. Snaith , “The Hospital Anxiety and Depression Scale,” Acta Psychiatrica Scandinavica 67, no. 6 (1983): 361–370. 6880820 10.1111/j.1600-0447.1983.tb09716.x

[27] R. C. Spiller , D. J. Humes , E. Campbell , et al., “The Patient Health Questionnaire 12 Somatic Symptom Scale as a Predictor of Symptom Severity and Consulting Behaviour in Patients With Irritable Bowel Syndrome and Symptomatic Diverticular Disease,” Alimentary Pharmacology and Therapeutics 32, no. 6 (2010): 811–820. 20629976 10.1111/j.1365-2036.2010.04402.x

[28] K. Kroenke , R. L. Spitzer , and J. B. Williams , “The PHQ‐15: Validity of a New Measure for Evaluating the Severity of Somatic Symptoms,” Psychosomatic Medicine 64, no. 2 (2002): 258–266. 11914441 10.1097/00006842-200203000-00008

[29] J. S. Labus , R. Bolus , L. Chang , et al., “The Visceral Sensitivity Index: Development and Validation of a Gastrointestinal Symptom‐Specific Anxiety Scale,” Alimentary Pharmacology and Therapeutics 20, no. 1 (2004): 89–97. 10.1111/j.1365-2036.2004.02007.x 15225175

[30] S. J. Lewis and K. W. Heaton , “Stool Form Scale as a Useful Guide to Intestinal Transit Time,” Scandinavian Journal of Gastroenterology 32, no. 9 (1997): 920–924. 9299672 10.3109/00365529709011203

[31] C. J. Black , Y. Yiannakou , E. Guthrie , R. West , L. A. Houghton , and A. C. Ford , “Longitudinal Follow‐up of a Novel Classification System for Irritable Bowel Syndrome: Natural History and Prognostic Value,” Alimentary Pharmacology & Therapeutics 53, no. 10 (2021): 1126–1137. 33705578 10.1111/apt.16322

[32] A. J. Irvine , W. D. Chey , and A. C. Ford , “Screening for Celiac Disease in Irritable Bowel Syndrome: An Updated Systematic Review and Meta‐Analysis,” American Journal of Gastroenterology 112, no. 1 (2017): 65–76. 27753436 10.1038/ajg.2016.466

[33] L. Wedlake , R. A'Hern , D. Russell , K. Thomas , J. R. Walters , and H. J. Andreyev , “Systematic Review: The Prevalence of Idiopathic Bile Acid Malabsorption as Diagnosed by SeHCAT Scanning in Patients With Diarrhoea‐Predominant Irritable Bowel Syndrome,” Alimentary Pharmacology and Therapeutics 30 (2009): 707–717. 19570102 10.1111/j.1365-2036.2009.04081.x

[34] K. M. Fairbrass , S. J. Costantino , D. J. Gracie , and A. C. Ford , “Prevalence of Irritable Bowel Syndrome‐Type Symptoms in Patients With inflammatory Bowel Disease in Remission: A Systematic Review and Meta‐Analysis,” Lancet Gastroenterology & Hepatology 5 (2020): 1053–1062. 33010814 10.1016/S2468-1253(20)30300-9

[35] C. Hookway , S. Buckner , P. Crosland , and D. Longson , “Irritable Bowel Syndrome in Adults in Primary Care: Summary of Updated NICE Guidance,” BMJ (Clinical Research Ed.) 350 (2015): h701. 10.1136/bmj.h701 25716701

[36] D. H. Vasant , P. A. Paine , C. J. Black , et al., “British Society of Gastroenterology Guidelines on the Management of Irritable Bowel Syndrome,” Gut 70, no. 7 (2021): 1214–1240. 33903147 10.1136/gutjnl-2021-324598

[37] L. Agreus , K. Svardsudd , N. J. Talley , M. P. Jones , and G. Tibblin , “Natural History of Gastroesophageal Reflux Disease and Functional Abdominal Disorders: A Population‐Based Study,” American Journal of Gastroenterology 96, no. 10 (2001): 2905–2914. 11693325 10.1111/j.1572-0241.2001.04680.x

[38] O. S. Palsson , J. Baggish , and W. E. Whitehead , “Episodic Nature of Symptoms in Irritable Bowel Syndrome,” American Journal of Gastroenterology 109, no. 9 (2014): 1450–1460. 24980882 10.1038/ajg.2014.181

[39] L. Agreus , K. Svardsudd , O. Nyren , and G. Tibblin , “Irritable Bowel Syndrome and Dyspepsia in the General Population: Overlap and Lack of Stability Over Time,” Gastroenterology 109, no. 3 (1995): 671–680. 7657095 10.1016/0016-5085(95)90373-9

[40] A. L. Engsbro , M. Simren , and P. Bytzer , “Short‐Term Stability of Subtypes in the Irritable Bowel Syndrome: Prospective Evaluation Using the Rome III Classification,” Alimentary Pharmacology & Therapeutics 35, no. 3 (2012): 350–359. 22176384 10.1111/j.1365-2036.2011.04948.x

[41] R. E. Williams , C. L. Black , H. Y. Kim , et al., “Stability of Irritable Bowel Syndrome Using a Rome II‐Based Classification,” Alimentary Pharmacology & Therapeutics 23, no. 1 (2006): 197–205. 16393298 10.1111/j.1365-2036.2006.02723.x

[42] L. B. Olafsdottir , H. Gudjonsson , H. H. Jonsdottir , and B. Thjodleifsson , “Stability of the Irritable Bowel Syndrome and Subgroups as Measured by Three Diagnostic Criteria ‐ a 10‐Year Follow‐up Study,” Alimentary Pharmacology & Therapeutics 32, no. 5 (2010): 670–680. 20604748 10.1111/j.1365-2036.2010.04388.x

