# Supplementary material for: Stability of Classification Systems for Irritable Bowel Syndrome
Source: Aliment Pharmacol Ther. 2025 Dec 25;63(8):1132–9. doi: 10.1111/apt.70503 (PMC13021291; doi:10.1111/apt.70503)
Supplement: Supplementary file 1 — Table S1: Variables used to assign cluster membership in the latent class analysis. Table S2: Demographic data, psychological characteristics and classification of individuals with Rome IV‐defined IBS at baseline followed up successfully at 12 months versus individuals with Rome IV‐defined IBS at baseline not followed up at 12 months. Table S3: Stability of IBS according to the Bristol stool form scale in 352 individuals with Rome IV IBS at baseline. Table S4: Stability of IBS according to most troublesome symptom in 352 individuals with Rome IV IBS at baseline. Table S5: Stability of IBS according to clusters based on both gastrointestinal and psychological symptoms in 352 individuals with Rome IV IBS at baseline. Table S6: Stability of IBS according to clusters based on the degree of psychological burden in 352 individuals with Rome IV IBS at baseline. Figure S1: Profiles of the seven clusters. [file APT-63-1132-s001.docx]

**Supplementary Table 1. Variables Used to Assign Cluster Membership in the Latent Class Analysis.**

|  | **Variable** | **Type of variable** | **Scale of Measurement** | **Reason for including in the model** |
| --- | --- | --- | --- | --- |
| **Gastrointestinal Symptoms** | Frequency of abdominal pain (or discomfort*) anywhere in the abdomen in past 3 months | Ordinal | 9-point scale from “Never” (0) to “Multiple times per day or all the time” (8) | All of these variables for quantifying gastrointestinal symptoms were taken from Rome Foundation questionnaires. These are the recognised “gold standard” for diagnosing IBS and are widely used. |
|  | Frequency of abdominal pain being closely related to a bowel movement | Ordinal | 11-point scale from “0%” (never) to “100%” (always) |  |
|  | Frequency with which abdominal pain improved or resolved following a bowel movement | Ordinal | 11-point scale from “0%” (never) to “100%” (always) |  |
|  | Frequency with which stools became softer or harder than usual in association with abdominal pain | Ordinal | 11-point scale from “0%” (never) to “100%” (always) |  |
|  | Frequency with which stools became more or less frequent than usual in association with abdominal pain | Ordinal | 11-point scale from “0%” (never) to “100%” (always) |  |
|  | Frequency with which abdominal pain started or got worse after a meal | Ordinal | 11-point scale from “0%” (never) to “100%” (always) |  |
|  | Frequency with which abdominal pain restricted usual activities | Ordinal | 11-point scale from “0%” (never) to “100%” (always) |  |
|  | Frequency of hard or lumpy stools in last 3 months | Ordinal | 5-point scale from “0%” (never or rarely) to “100%” (always) |  |
|  | Frequency of loose, mushy, or watery stools in the last 3 months | Ordinal | 5-point scale from “0%” (never or rarely) to “100%” (always) |  |
|  | Frequency of faecal urgency over last 3 months | Ordinal | 9-point scale from “Never” (0) to “Multiple times per day or all the time” (8) |  |
|  | Frequency of faecal incontinence over last 3 months | Ordinal | 9-point scale from “Never” (0) to “Multiple times per day or all the time” (8) |  |
|  | Frequency of abdominal bloating or distension over last 3 months | Ordinal | 9-point scale from “Never” (0) to “Multiple times per day or all the time” (8) |  |
| **Extraintestinal Symptoms** | All individual items of the PHQ-12 and the frequency experienced in the last 4 weeks:  Back pain  Arm, leg, joint pain  Period pain/period problems  Headaches  Chest pain  Dizziness  Fainting spells  Heart pounding/racing  Shortness of breath  Pain/problems during sex  Feeling tired or low in energy  Trouble sleeping | Ordinal | 3-point scale: “Never” (0), “A little” (1), or “A lot” (2) | Reporting symptoms referable to multiple body systems, also referred to as somatisation, is recognised as being associated with IBS and other disorders of gut-brain interaction. The PHQ-12 questionnaire is a widely used and validated method for measuring this. |
| **Mood** | Presence of anxiety, as measured by the total score of the HADS-Anxiety questionnaire | Ordinal | 3-point scale: normal (0), borderline (1), or abnormal (2) | Abnormal mood is well-recognised as being an important factor in IBS. The HADS questionnaire for quantifying the presence of anxiety and/or depression are widely used and validated for this purpose. |
|  | Presence of depression, as measured by the total score of HADS-Depression questionnaire | Ordinal | 3-point scale: normal (0), borderline (1), or abnormal (2) |  |

HADS; hospital anxiety and depression scale, PHQ-12; patient health questionnaire-12.

**Supplementary Table 2. Demographic Data, Psychological Characteristics, and Classification of Individuals with Rome IV-defined IBS at Baseline Followed Up Successfully at 12 Months Versus Individuals with Rome IV-defined IBS at Baseline Not Followed Up at 12 Months.**

|  | **Rome IV-defined IBS at baseline followed up successfully at 12 months**  **(n = 352)** | **Rome IV-defined IBS at baseline not followed up at 12 months**  **(n = 400)** | ***p* value*** |
| --- | --- | --- | --- |
| **Mean age at baseline (SD)** | 48.6 (14.7) | 42.4 (14.3) | <0.001 |
| **Female (%)** | 302 (85.8) | 353 (88.3) | 0.32 |
| **White Caucasian ethnicity (%)** | 345 (98.0) | 384 (96.0) | 0.65 |
| **Married or cohabiting at baseline (%)** | 232 (65.9) | 255 (63.7) | 0.086 |
| **University or postgraduate level of education at baseline (%)** | 186 (25.85) | 106 (26.5) | 0.50 |
| **Annual income of ≥£30,000 at baseline (%)** | 31 (25.6) | 86 (31.3) | 0.74 |
| **Smoker at baseline (%)** | 23 (6.5) | 59 (14.8) | <0.001 |
| **Alcohol user at baseline (%)** | 214 (60.8) | 225 (56.3) | 0.21 |
| **Duration of IBS symptoms, in years, at baseline (%)**  1  2  3  4  5  >5 | 11 (3.1)  21 (6.0)  27 (7.7)  12 (3.4)  17 (4.8)  264 (75.0) | 14 (3.5)  20 (5.0)  27 (6.8)  21(5.3)  21 (5.3)  297 (74.3) | 0.14 |
| **IBS after acute enteric infection at baseline (%)** | 49 (13.9) | 42 (10.5) | 0.11 |
| **Seen a primary care physician with IBS in last 12 months at baseline (%)** | 128 (43.5) | 166 (56.5) | 0.15 |
| **Seen a gastroenterologist with IBS in last 12 months at baseline (%)** | 75 (21.3) | 72 (18.0) | 0.25 |
| **Symptom severity on IBS-SSS at baseline (%)**  Remission  Mild  Moderate  Severe | 3 (0.9)  42 (11.9)  145 (41.2)  162 (46.0) | 4 (1.0)  44 (11.0)  155 (38.8)  197 (49.3) | 0.83 |
| **HADS-A category at baseline (%)**  Normal  Borderline abnormal  Abnormal | 102 (29.0)  83 (23.6)  167 (47.4) | 98 (24.5)  91 (22.8)  211 (52.8) | 0.28 |
| **HADS-D category at baseline (%)**  Normal  Borderline abnormal  Abnormal | 189 (53.7)  82 (23.3)  81 (23.0) | 215 (53.8)  83 (20.8)  102 (25.5) | 0.60 |
| **PHQ-12 severity at baseline (%)**  Minimal  Low  Moderate  High | 18 (5.1)  90 (25.6)  143 (40.6)  101(28.7) | 18 (4.5)  86 (21.5)  164 (41.0)  132 (33.0) | 0.46 |
| **Gastrointestinal symptom-specific anxiety on VSI at baseline (%)**  Low  Medium  High | 129 (36.6)  107 (30.4)  116 (33.0) | 118 (29.5)  140 (35.0)  142 (35.5) | 0.11 |
| **IBS subtype at baseline (%)**  IBS-C  IBS-D  IBS-M  IBS-U | 58 (16.5)  141 (40.1)  148 (42.0)  5 (1.4) | 78 (19.5)  165 (41.3)  153 (38.3)  4 (1.0) | 0.43 |
| **Most troublesome symptom at baseline (%)**  Abdominal pain  Constipation  Diarrhea  Abdominal bloating or distension  Urgency | 76 (21.6)  28 (8.0)  59 (16.8)  96 (27.3)  93 (26.4) | 93 (23.3)  25 (6.3)  58 (14.5)  122 (30.5)  102 (25.5) | 0.67 |
| **Cluster membership at baseline (%)**  1. Diarrhoea and urgency, low psychological burden  2. Low overall gastrointestinal symptom severity, high psychological burden  3. Low overall gastrointestinal symptom severity, low psychological burden  4. Diarrhoea, abdominal pain, and urgency, high psychological burden  5. Constipation, abdominal pain, and bloating, high psychological burden  6. High overall gastrointestinal symptom severity, high psychological burden  7. Constipation and bloating, low psychological burden | 73 (20.7)  94 (26.7)  63 (17.9)  61 (17.3)  12 (3.4)  28 (8.0)  21 (6.0) | 67 (16.8)  101 (25.3)  80 (20.0)  86 (21.5)  12 (3.0)  28 (7.0)  26 (6.5) | 0.64 |
| **High psychological burden cluster at baseline (%)** | 195 (55.4) | 227 (56.8) | 0.71 |

**p* value for independent samples *t*-test for continuous data and Pearson χ^2^ for comparison of categorical data.

IBS-C; IBS with constipation, IBS-D; IBS with diarrhoea, IBS-M; IBS with mixed bowel habits, IBS-U; IBS unclassified, HADS; hospital anxiety and depression scale, IBS-SSS; irritable bowel syndrome severity scoring system, PHQ-12; patient health questionnaire-12, VSI; visceral sensitivity index.

**Supplementary Table 3. Stability of IBS According to the Bristol Stool Form Scale in 352 Individuals with Rome IV IBS at Baseline.**

|  | **IBS-C at 12-month follow-up (%)** | **IBS-D at 12-month follow-up (%)** | **IBS-M at 12-month follow-up (%)** | **IBS-U at 12-month follow-up (%)** |
| --- | --- | --- | --- | --- |
| **IBS-C at baseline (n=58)** | 39 (67.2) | 2 (3.4) | 14 (24.1) | 3 (5.2) |
| **IBS-D at baseline**  **(n=141)** | 5 (3.5) | 117 (83.0) | 18 (12.8) | 1 (0.7) |
| **IBS-M at baseline (n=148)** | 20 (13.5) | 30 (20.3) | 98 (66.2) | 0 (0.0) |
| **IBS-U at baseline (n=5)** | 0 (0.0) | 1 (20.0) | 3 (60.0) | 1 (20.0) |

Kappa = 0.57

**Supplementary Table 4. Stability of IBS According to Most Troublesome Symptom in 352 Individuals with Rome IV IBS at Baseline.**

|  | **Abdominal pain at 12-month follow-up (%)** | **Constipation at 12-month follow-up (%)** | **Diarrhoea at 12-month follow-up (%)** | **Bloating or distension at 12-month follow-up (%)** | **Urgency at 12-month follow-up (%)** |
| --- | --- | --- | --- | --- | --- |
| **Abdominal pain at baseline (n=76)** | 49 (64.5) | 4 (5.3) | 6 (7.9) | 11 (14.5) | 6 (7.9) |
| **Constipation at baseline**  **(n=28)** | 2 (7.1) | 19 (67.9) | 0 (0.0) | 7 (25.0) | 0 (0.0) |
| **Diarrhoea at baseline (n=59)** | 5 (8.5) | 1 (1.7) | 28 (47.5) | 5 (8.5) | 20 (33.9) |
| **Bloating or distension at baseline (n=96)** | 14 (14.6) | 6 (6.3) | 4 (4.2) | 65 (67.7) | 7 (7.3) |
| **Urgency at baseline (n=93)** | 8 (8.6) | 3 (3.2) | 21 (22.6) | 12 (12.9) | 49 (52.7) |

Kappa = 0.48

**Supplementary Table 5. Stability of IBS According to Clusters Based on Both Gastrointestinal and Psychological Symptoms in 352 Individuals with Rome IV IBS at Baseline.**

|  | **Cluster 1: Diarrhoea and urgency with low psychological burden at 12-month follow-up (%)** | **Cluster 2: Low bowel symptom severity with abdominal pain and high psychological burden at 12-month follow-up (%)** | **Cluster 3: Low overall gastrointestinal symptom severity with low psychological burden at 12-month follow-up (%)** | **Cluster 4: Diarrhoea, abdominal pain, and urgency with high psychological burden at 12-month follow-up (%)** | **Cluster 5: Constipation, abdominal pain, and bloating with high psychological burden at 12-month follow-up (%)** | **Cluster 6: High overall gastrointestinal symptom severity with high psychological burden at 12-month follow-up (%)** | **Cluster 7: Constipation and bloating with low psychological burden at 12-month follow-up (%)** |
| --- | --- | --- | --- | --- | --- | --- | --- |
| **Cluster 1: Diarrhoea and urgency with low psychological burden at baseline (n=73)** | 30 (41.1) | 11 (15.1) | 17 (23.3) | 13 (17.8) | 0 (0.0) | 1 (1.4) | 1 (1.4) |
| **Cluster 2: Low bowel symptom severity with abdominal pain and high psychological burden at baseline (n=94)** | 10 (10.6) | 49 (52.1) | 14 (14.9) | 10 (10.6) | 2 (2.1) | 4 (4.3) | 5 (5.3) |
| **Cluster 3: Low overall gastrointestinal symptom severity with low psychological burden at baseline (n=63)** | 14 (22.2) | 5 (7.9) | 41 (65.1) | 2 (3.2) | 0 (0.0) | 0 (0.0) | 1 (1.6) |
| **Cluster 4: Diarrhoea, abdominal pain, and urgency with high psychological burden at baseline (n=61)** | 8 (13.1) | 7 (11.5) | 7 (11.5) | 29 (47.5) | 2 (3.3) | 7 (11.5) | 1 (1.6) |
| **Cluster 5: Constipation, abdominal pain, and bloating with high psychological burden at baseline (n=12)** | 0 (0.0) | 3 (25.0) | 0 (0.0) | 1 (8.3) | 5 (41.7) | 1 (8.3) | 2 (16.7) |
| **Cluster 6: High overall gastrointestinal symptom severity with high psychological burden at baseline (n=28)** | 0 (0.0) | 8 (28.6) | 0 (0.0) | 3 (10.7) | 2 (7.1) | 15 (53.6) | 0 (0.0) |
| **Cluster 7: Constipation and bloating with low psychological burden at baseline (n=21)** | 3 (14.3) | 5 (23.8) | 5 (23.8) | 1 (4.8) | 0 (0.0) | 0 (0.0) | 7 (33.3) |

Kappa = 0.39

**Supplementary Table 6. Stability of IBS According to Clusters Based on the Degree of Psychological Burden in 352 Individuals with Rome IV IBS** **at Baseline.**

|  | **Low psychological burden cluster at 12-month follow-up (%)** | **High psychological burden cluster at 12-month follow-up (%)** |
| --- | --- | --- |
| **Low psychological burden cluster at baseline (n=157)** | 119 (75.8) | 38 (24.2) |
| **High psychological burden cluster at baseline (n=195)** | 47 (24.1) | 148 (75.9) |

Kappa = 0.51


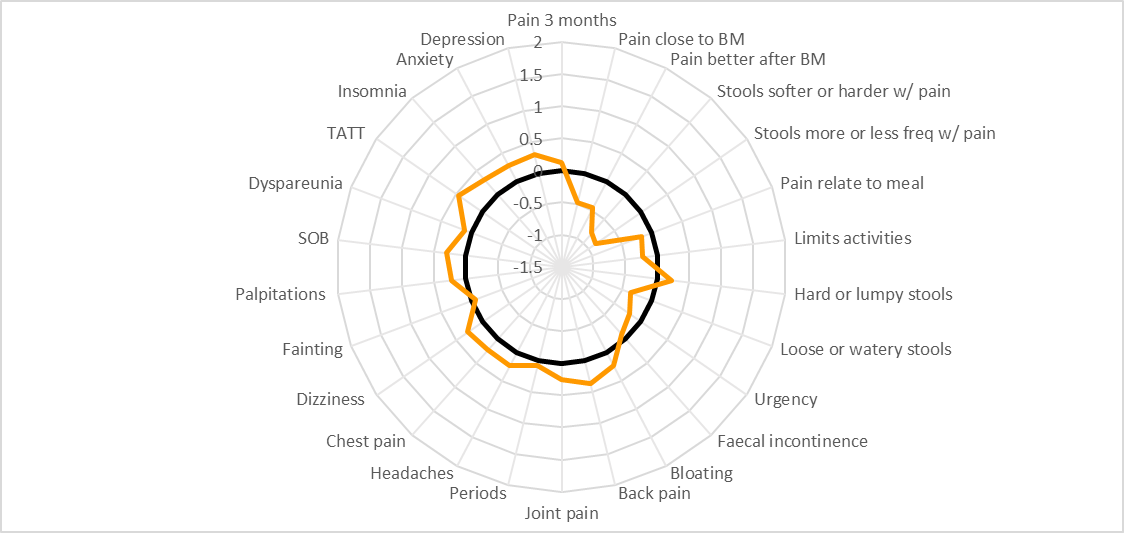

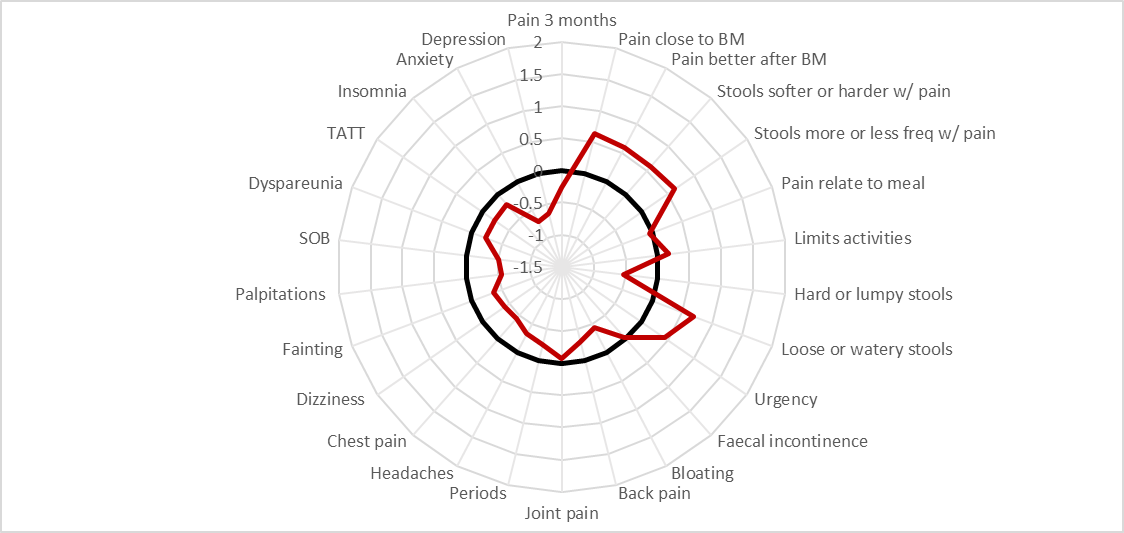
**Supplementary Figure 1. Profiles of the Seven Clusters.**


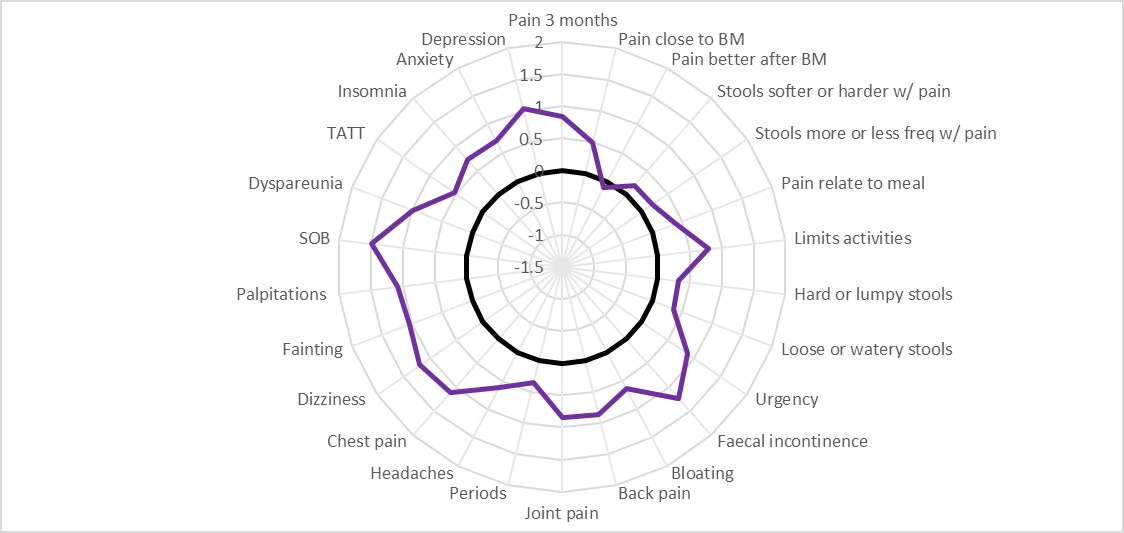

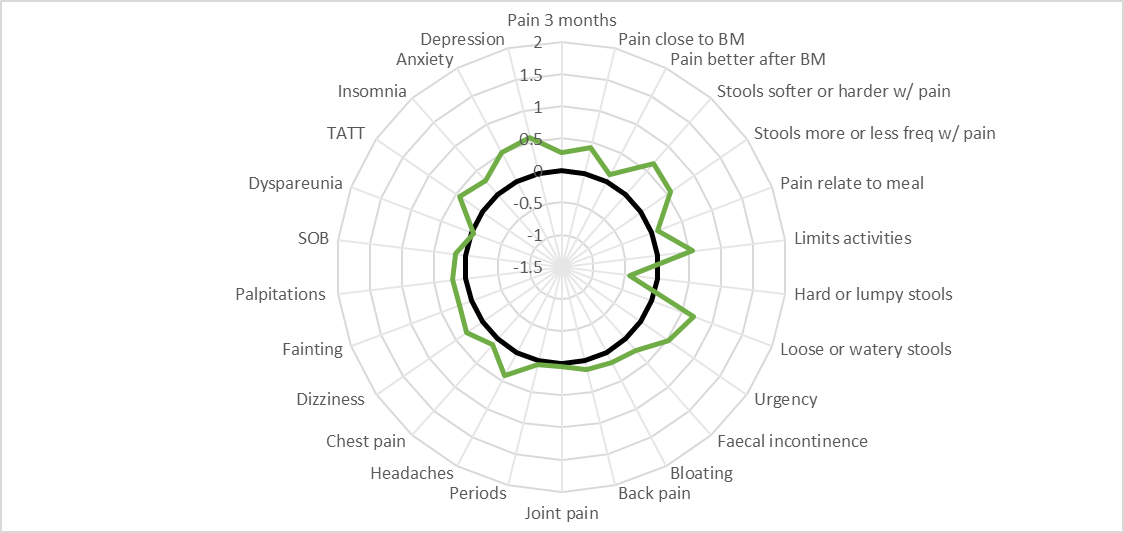

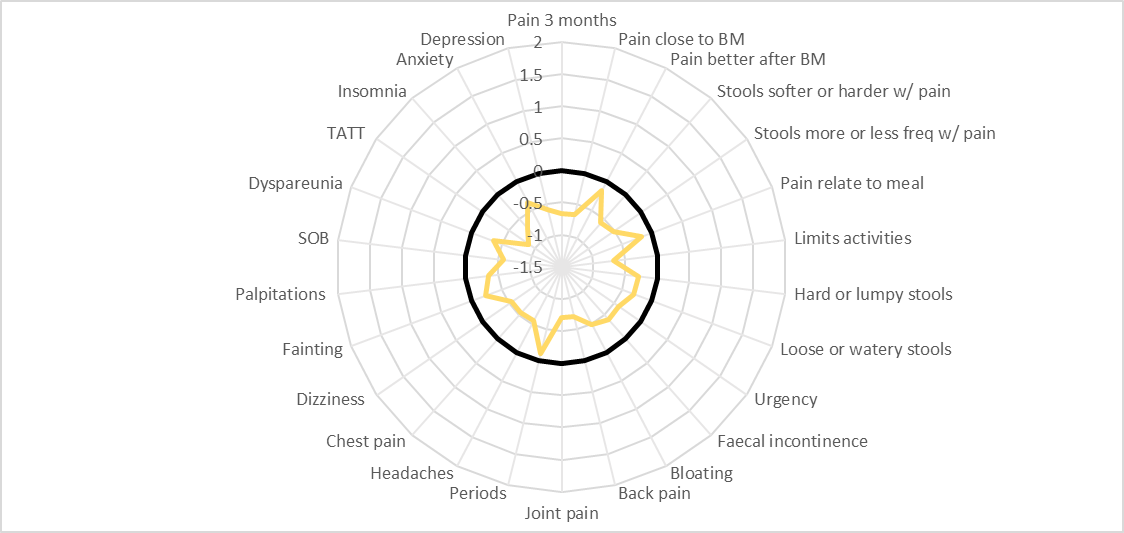


**B**

**A**


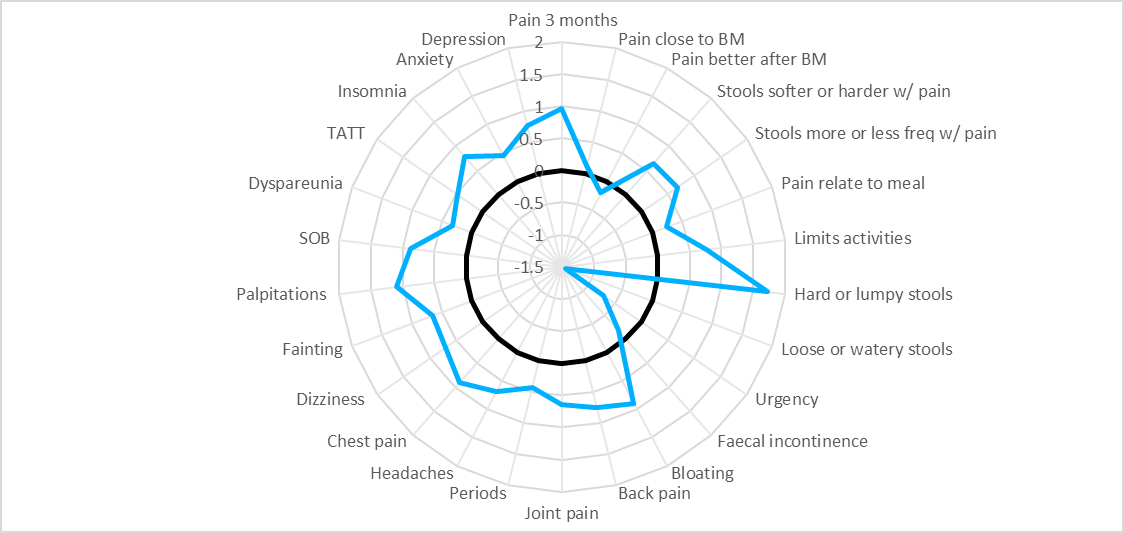


**C**

**D**


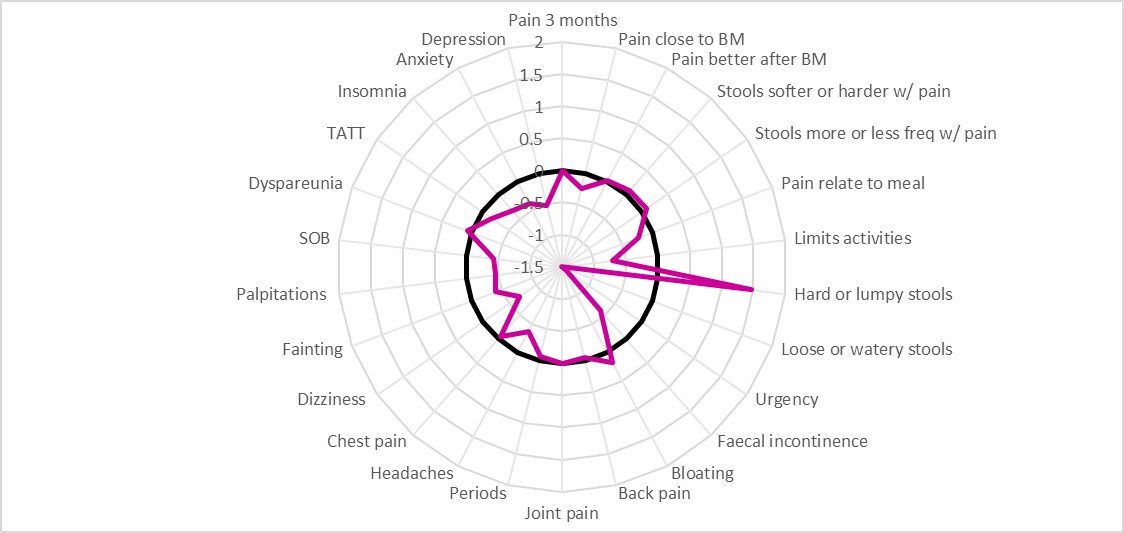


**F**

**E**

**G**

= adjusted cohort mean

1. Cluster 1: Diarrhoea and urgency with low psychological burden.
2. Cluster 2: Low bowel symptom severity with abdominal pain and high psychological burden.
3. Cluster 3: Low overall gastrointestinal symptom severity with low psychological burden.
4. Cluster 4: Diarrhoea, abdominal pain, and urgency with high psychological burden.
5. Cluster 5: Constipation, abdominal pain, and bloating with high psychological burden.
6. Cluster 6: High overall gastrointestinal symptom severity with high psychological burden.
7. Cluster 7: Constipation and bloating with low psychological burden.

BM: bowel movement; SOB: shortness of breath; TATT: tired all the time
